# Microneedle mediated intradermal delivery of adjuvanted recombinant HIV-1 CN54gp140 effectively primes mucosal boost inoculations

**DOI:** 10.1016/j.jconrel.2012.07.039

**Published:** 2012-09-28

**Authors:** Aditya Pattani, Paul F. McKay, Martin J. Garland, Rhonda M. Curran, Katarzyna Migalska, Corona M. Cassidy, R. Karl Malcolm, Robin J. Shattock, Helen O. McCarthy, Ryan F. Donnelly

**Affiliations:** a Queen's University Belfast, School of Pharmacy, Belfast BT9 7BL, UK; b Imperial College London, Department of Infectious Diseases, Division of Medicine, Norfolk Place, London W2 1PG, UK

**Keywords:** HIV, Vaccination, Microneedle, Intranasal, Subcutaneous

## Abstract

Dissolving polymeric microneedle arrays formulated to contain recombinant CN54 HIVgp140 and the TLR4 agonist adjuvant MPLA were assessed for their ability to elicit antigen-specific immunity. Using this novel microneedle system we successfully primed antigen-specific responses that were further boosted by an intranasal mucosal inoculation to elicit significant antigen-specific immunity. This prime-boost modality generated similar serum and mucosal gp140-specific IgG levels to the adjuvanted and systemic subcutaneous inoculations. While the microneedle primed groups demonstrated a balanced Th1/Th2 profile, strong Th2 polarization was observed in the subcutaneous inoculation group, likely due to the high level of IL-5 secretion from cells in this group. Significantly, the animals that received a microneedle prime and intranasal boost regimen elicited a high level IgA response in both the serum and mucosa, which was greatly enhanced over the subcutaneous group. The splenocytes from this inoculation group secreted moderate levels of IL-5 and IL-10 as well as high amounts of IL-2, cytokines known to act in synergy to induce IgA. This work opens up the possibility for microneedle-based HIV vaccination strategies that, once fully developed, will greatly reduce risk for vaccinators and patients, with those in the developing world set to benefit most.

## Graphical abstract

**Figure:**
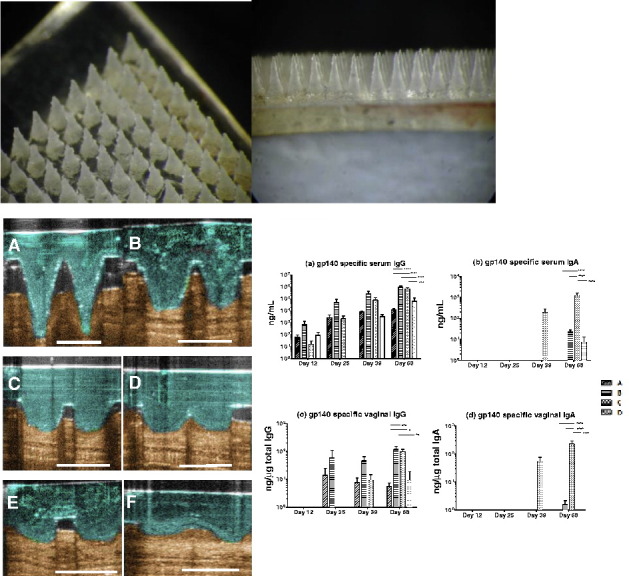

## 1 Introduction

Mucosal immune responses are likely to be critical in preventing transmission of many sexually or respiratorily transmitted diseases, including HIV and influenza. While a large number of mucosal vaccine immunization modalities have been tested in preclinical and clinical studies, it has been shown that the vaginal mucosa, the portal of entry for many sexually transmitted pathogens in women, is particularly refractory to the elicitation of immune responses [1]. The non-inductive nature of the vaginal mucosa is therefore a major barrier enabling women to protect themselves by vaccination. Our recent studies in mice have indicated that antigen-specific vaginal immune responses can be elicited with a sub-cutaneous (SC) vaccine prime followed with intravaginal (Ivag) boosting [2]. However, administration of SC injections requires trained medical staff and correct and safe disposal of used needles, both of which are difficult to achieve in resource poor areas. Alternative delivery and formulation technologies that can be self-applied may overcome these barriers to effective mucosal protection.

**Fig. 1:**
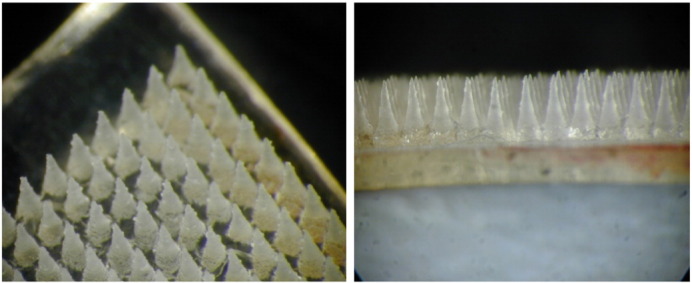
The structure of a MN array (placebo, Gantrez® based soluble microneedles of the type and geometry used in this study, mean height of each microneedle ~ 600 μm) — top view (left) and side view (right).

Microneedles (MNs) are sub-millimetre structures designed to pierce the skin's *stratum corneum* barrier and deliver active agent(s) into the epidermal or dermal compartments [3]. They are usually designed as arrays (Fig. 1) to provide a large number of distinct skin penetrations within a small surface area and therefore deliver sufficiently large doses for clinical efficacy. MNs are an attractive antigen delivery system as the vaccine formulation is made readily available to immune responsive antigen presenting cells (APCs) in the skin, such as Langerhans cells in the epidermis and dendritic cells in the dermis [4–6]. Compared to conventional parenteral routes (e.g. intramuscular, subcutaneous), dose sparing for vaccination has been observed for MNs [7,8]. Recently, MN administration of an influenza vaccine has been reported to offer protection in the mouse model at least equivalent to that of a conventional intramuscular injection [9]. Importantly, the MNs developed by our group rapidly dissolve in skin interstitial fluid and are therefore self-disabling and cannot be re-used after removal, with the added benefit that disposal issues associated with conventional needles are also overcome. These MNs deliver a specific dose of vaccine antigen over a relatively short period of time, both variables that are easily altered.

In the current study we assessed the feasibility of a microneedle (MN) approach designed to rapidly dissolve and deliver a stable trimeric recombinant HIV-1 CN54 clade C gp140 envelope protein to immune responsive cells and initiate antigen-specific immune responses. The clade C HIV-1 subtype has a high global prevalence, and this antigen candidate has already been evaluated in several pre-clinical studies [2,10–13], a Phase I human clinical trial [1], and is being further evaluated in ongoing clinical studies. The novel MN system was formed by micromoulding a mucoadhesive and vaccine antigen loaded copolymer. We further determined if the vaccine generated immune responses in MN-primed animals were subsequently boosted by topical mucosal vaccination. To the best of our knowledge, this is the first reported evaluation of the use of a MN system for HIV immunization. The candidate vaccine antigen CN54 gp140 has previously been shown to be poorly immunogenic when applied to the vaginal mucosae [2,12,13]. Therefore, monophosphoryl lipid A (MPLA) was used as an adjuvant in order to enhance the immune response. The objectives of the study were (i) to assess a novel antigen/adjuvant-loaded and rapidly dissolving MN array device as a tool for the non-invasive needle-free intradermal delivery of molecules, (ii) to determine if these vaccine loaded MNs can be used to effectively prime and/or boost a gp140-specific antibody response, and (iii) to determine if the vaccine-elicited immune responses had any potentially important characteristics that could improve vaccine efficacy.

## 2 Materials and methods

HIV-1 CN54gp140 (gp120 plus the ectodomain of gp41) was encoded by the CN54gp140REKE HIV-1 envelope gene cassette derived from the clade-C/B′ HIV-1 molecular clone p97CN54 of Chinese origin developed by Wolf and Wagner, University of Regensburg, Germany [14,15]. CN54gp140 was produced as a recombinant product in CHO cells by S. Jeffs, Imperial College, London, and manufactured to GMP specification by Polymun Scientific Immunbiologische Forschung GmbH, Austria. Gantrez® AN-139 (a copolymer of methylvinylether and maleic anhydride) was obtained from ISP Co. Ltd., UK. 3,3′,5,5′-Tetramethylbenzidine peroxidase substrate (TMB/E) was obtained from Cygnus Technologies Inc., USA. Polysorbate 80, concanavalin A, sodium hydroxide and bovine serum albumin were purchased from Sigma-Aldrich, UK. Anti-mouse Ig kappa and lambda light chain specific antibodies were obtained from Serotec, UK. HRP-conjugated anti-mouse IgG, IgG1, IgG2a and biotinylated goat anti-mouse IgA were obtained from Cambridge Bioscience Ltd., UK. HRP-conjugated streptavidin was purchased from R&D Systems Europe Ltd., USA. Depo-Provera® injection (medroxyprogesterone acetate 150 mg/mL, Pharmacia Ltd., UK.) was kindly gifted by Belfast City Hospital. Monophosphoryl lipid A (MPLA) was obtained from InvivoGen, USA. General use protease inhibitor cocktail was obtained from Sigma, UK and reconstituted to 10 × concentration. This was supplemented with 10 mM phenylmethanesulfonyl fluoride (PMSF) immediately prior to use. Nunc Maxisorp 96 well microplates were obtained from Nunc, Denmark. ELISpot^PLUS^ B cell ELISpot kits were obtained from Mabtech, Sweden and a Bio-plex Pro™ mouse cytokine Th1/Th2 assay kit was obtained from Bio-Rad, USA. The water used for formulation and analysis was deionised using a Milli-Q® (Millipore, Ireland) system to a resistivity of 18.2 MΩ·cm. All in vivo procedures were carried out in compliance with the UK animal (scientific procedures) act 1986 and associated codes of practice for the housing and care of animals.

### 2.1 Preparation of microneedles

The laser engineered micromoulding technique was used for the purpose of preparation of soluble MNs, as reported previously [16]. Each MN array (comprising 361 needles of 600 μm height, 300 μm base width, 50 μm interspacing on a 1 cm^2^ base support with a total theoretical mass of 5.10 mg and a backing layer) contained an antigen (10 μg) and an adjuvant (20 μg). MNs were micromoulded from aqueous blends of the mucoadhesive copolymer Gantrez® AN-139 in two steps, resulting in an antigen/adjuvant containing layer, composed of the needles and a thin base plate, to which a drug-free backing layer was added after drying. In production of the drug containing section, the requisite antigen and adjuvant were added to Gantrez® (30% solution) which had been neutralised with sodium hydroxide to pH 7.4. Phosphate buffered saline pH 7.4 and Polysorbate 80 were added to produce a homogeneous mixture, which was poured into the silicone micromoulds and centrifuged (3000 rpm, 30 min). The arrays were then dried under ambient temperature for 48 h with centrifugation (3000 rpm for two 30 min periods) which allowed for evaporation of the aqueous phase, leaving the polymer matrix encasing the antigen/adjuvant at the requisite loading. At this time, another portion of neutralised Gantrez® was added to the moulds. The arrays were centrifuged (3000 rpm, 15 min) and dried for a further 48 h. The side walls were removed prior to needle application. These MNs are considered to be ‘soluble’ i.e. the polymeric needles dissolve in contact with the interstitial fluid following administration to the skin [16]. In addition, the soluble MN arrays, post immunization do not have any ‘sharps’, obviating the need for specific disposal procedures.

### 2.2 Optical coherence tomographic assessment of microneedle penetration, and subsequent dissolution, into murine ear skin in vivo

The penetration characteristics, and subsequent in-skin dissolution kinetics, of non-antigen/adjuvant loaded PMVE/MA MN arrays following manual application, using gentle finger pressure by the same trained, experienced operator, to the ear of anaesthetised mice in vivo were determined using optical coherence tomography (VivoSight® high resolution OCT scanner with handheld probe (Michelson Diagnostics Ltd., Kent, UK)), as described previously [17]. The swept-source Fourier domain OCT system has a laser centre wavelength of 1305.0 ± 15.0 nm, facilitating real time high resolution imaging of the upper skin layers (7.5 μm lateral and 10.0 μm vertical resolution). The skin was scanned at a rate of up to 15 B-scans (2D cross-sectional scans) per second (scan width = 2.0 mm). 2D images were analysed using the imaging software ImageJ®. The scale of the image files obtained was 1 pixel = 4.2 μm, thus allowing accurate measurements of the depth of MN penetration, the width of the pore created, and the distance between the MN base plate and the *stratum corneum*. To allow differentiation between MNs and skin layers false colours were applied using Ability Photopaint® Version 4.14. In all instances, experiments were performed in triplicate, and > 5 MNs were measured for each replicate.

### 2.3 Immunization

Mice (BALB/c, females, Charles River, UK) 8–9 weeks old at the beginning of the experiment were used for the study. They were given a standard mouse diet ad-libitum and acclimatised for 1 week prior to beginning the experiment. All the mice were fitted with SC transponders for identification and weighed on all sampling days.

Mice received a prime (day 0) followed by three boosts at 14-day intervals (days 14, 28, 42, Table 1). MN arrays were administered to the ear using gentle thumb pressure following light anaesthesia (ketamine 100 mg/kg and xylazine 15 mg/kg administered intraperitoneally) and then secured to the ear overnight using an occlusive adhesive patch (Duro-Tack® pressure sensitive adhesive) followed by zinc oxide surgical tape. The ear was chosen as the site for MN application for two reasons. First, due to its close proximity to the draining neck lymph nodes, it was thought that this route of application might enable enhanced distribution to the lymphatic system following uptake by the high population of dendritic cells resident within the skin layers. Second, this application does not require hair removal procedures, which may trigger local immune responses and affect results. Intranasal (IN) doses were instilled into the nostrils using a standard Eppendorf® micropipette after ketamine/xylazine anaesthesia. Intravaginal (Ivag) doses were administered using a positive displacement pipette (Gilson Microman M100) and a sterile tip (CP100). To thin the vaginal epithelium and improve protein uptake specifically in vaginal boost group A, all mice (for uniformity) were treated with Depo-Provera® (SC, 2.5 mg in 100 μL PBS) 5 days prior to the first and third boosts. We also included a control SC route vaccination group. Blood samples were taken from the tail vein of the mice (Microvette® CB300Z tubes, Sarstedt, Germany) on days − 28, − 14, 12, 25, and 39, and by cardiac puncture on day 68, considering the prime as day 0. Blood samples were centrifuged following clotting for collection of sera. Vaginal lavages were also conducted on bleed days. Vaginal lavage for each animal was collected by flushing the vaginal lumen three times (25 μL, sterile PBS) using a positive displacement pipette and pooling the three eluates. Protease inhibitor cocktail (10 ×, 8 μL) was added to the vaginal elutes, which were then centrifuged at ~ 21,000 *g* (14,000 rpm, 4 °C, 10 min) to remove the mucus/cells. All samples were stored at − 80 °C until analysis.

### 2.4 Analysis of antibody levels

Binding antibodies against CN54gp140 in vaginal lavage and serum samples were measured using a quantitative ELISA. 96-Well maxisorp plates (Nunc, Denmark) were either coated with 50 μL of CN54gp140 antigen solution (5 μg/mL in sterile PBS) or 50 μL of a 1:3200 dilution of an equal mixture of anti-mouse Ig kappa and lambda light chain specific monoclonal antibodies (Serotec, UK) and then incubated O/N at 4 °C. Plates were then washed four times with PBS/0.05% Tween 20 (PBST) using an Aquamax 2000 automatic plate washer (Molecular Devices, UK) and blocked with 200 μL BSA (1%). Monoclonal murine IgG, IgG1, IgG2a or IgA standard immunoglobulins were used on each plate to quantify the murine anti-CN54gp140 specific antibodies. Experimental serum samples were diluted 1:100, 1:1000 and 1: 10,000 and lavage samples 1:10, 1:50 and 1:250 to ensure that the absorbance reading measured was within the linear range of the relevant standard curve. Bound or captured Ig was detected by incubation (1 h, 37 °C) with HRP-conjugated goat anti-mouse IgG, IgG1 or IgG2a (Cambridge BioScience Ltd., UK) and bound IgA was detected using biotinylated goat anti-mouse IgA (Cambridge BioScience Ltd., UK) and followed by streptavidin-HRP (R&D Systems Europe Ltd., UK). Plates were washed and developed with SureBlue TMB (50 μL; Insight Biotechnology Limited, UK) substrate and the reaction was terminated by the addition of 2 M H_2_SO_4_ (50 μL) and read at 450 nm using a plate reader. Vaginal lavage values were normalised against the total IgA or IgG measured in the same sample.

### 2.5 B cell ELISpots and cytokine analysis

Upon termination of experiments (day 68), mice were humanely culled and their spleens were aseptically removed and placed into ice-cold sterile RPMI-1640 supplemented with heat inactivated foetal bovine serum (10% v/v), l-glutamine (2 mM), penicillin (100 U/mL) and streptomycin (100 μg/mL), gentamicin (50 μg/mL), 2-mercaptoethanol (50 μM), HEPES (10 mM) and sodium pyrovate (1 mM). Single cell suspension was prepared by gently grinding the spleen on a 100 μm fine wire screen. The cells were centrifuged (2000 rpm) and RPMI removed. ACK RBC lysis buffer (5 mL) was added and allowed to act for 5 min. Then RPMI (5 mL) was added to stop the action of the ACK buffer, centrifuged as before and washed twice with RPMI. The spleen cells were all re-suspended in complete RPMI (2 mL/mouse), pooled, counted and adjusted to 2.5 × 10^6^ cells/mL (Z1 Coulter® particle counter, Beckman Coulter, UK).

The prepared mouse splenocyte cells (2.5 × 10^5^ total cells) were cultured unstimulated or in the presence of concanavalin A (2.5 μg/mL) or recombinant gp140 (10 μg/mL) for 48 h in 96 well plates. All the samples were analysed in quadruplicate. 100 μL of the cell suspension was removed and frozen (− 80 °C) for cytokine analysis. For the analysis of cytokines, the sample tubes were thawed and centrifuged to remove any cells or cellular debris and the supernatant was analysed by a multiplexed cytokine assay using a mouse Th1/Th2 multiplex panel (BioRad). Assays, using 50 μL of the prepared stimulated cell supernatants were carried out for IL-2, IL-12 p70, IFN-γ and TNF-α (Th1 cytokines), IL-4, IL-5 and IL-10 (Th2 cytokines) and GM-CSF (common to both sub-sets) using a Luminex 100 IS system (Luminex, USA) with IS xMAP 2.3 software. Standards supplied with the kit were used for the assay, each analyte having a different sensitivity range that is described within the manual for the kit but all our test samples fell within the range of the standard curve. The data were baseline corrected using the data from the unstimulated control cells (n = 3). The controls always secreted very low levels of cytokines and where the levels in the control (all replicates) were below the range of the standard curve, a value of zero was used, considering the sensitivity of the assay.

The spleen cell suspension (100 μL) adjusted to 2.5 × 10^6^ cells/mL was also used for B cell ELISpots (IgA) as per manufacturer's instructions. The cells were cultured overnight on an ELISpot plate previously coated with gp140 prepared in DPBS (100 μL, 10 μg/mL). Total IgA detection was used as a control. The plates were then developed and read using AID iSpot reader equipped with AID iSpot version 6.0 software (Autoimmun Diagnostika, GmbH, Germany). The data was baseline corrected using blank wells that had been developed using the same protocol.

### 2.6 Statistical analysis

Two-way repeated measures ANOVA (with Bonferroni's post hoc test) was used for analysing the antibody results. Kruskal Wallis test with Dunn's post hoc test was used for the analysis of cytokine data. The tests were carried out using Graphpad® Prism software. Confidence limits of 95% were used to determine statistical significance.

## 3 Results

### 3.1 MNs dissolve and deliver vaccine components rapidly in vivo

**Fig. 2:**
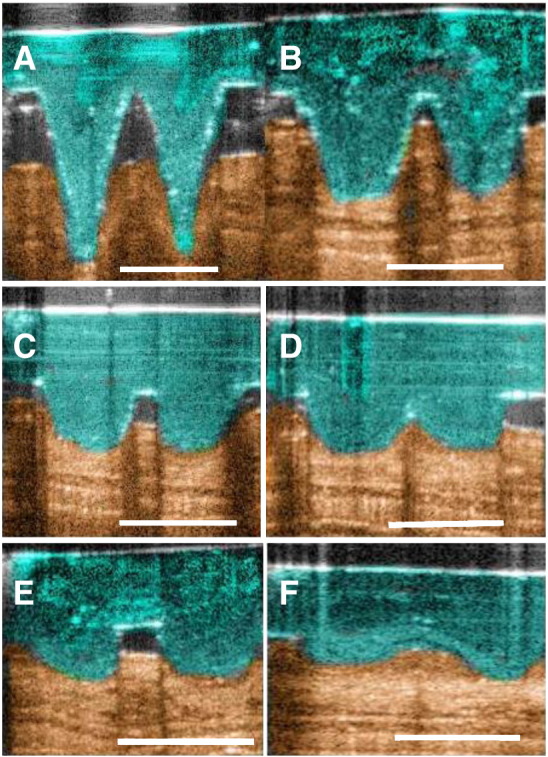
Optical coherence tomographic assessment, in real time and in situ, of PMVE/MA MN dissolution following insertion into ear of mice in vivo. (A) 0 min, (B) 2 min, (C) 3 min, (D) 5 min, (E) 10 min and (F) 15 min after insertion. Scale bar represents a length of 300 μm.

**Fig. 3:**
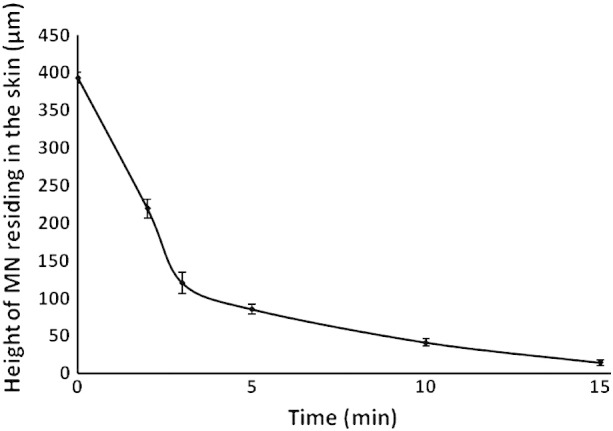
In-skin dissolution profile of PMVE/MA MNs following insertion into mice ear in vivo. (Means ± SD, n = 15).

The depth and reproducibility of in vivo MN penetration into murine ear skin were evaluated using optical coherence tomography (OCT) following manual application (Table 2 and Figs. 2, 3). The depth of MN penetration was approximately 400 μm, easily reaching the dermis within the mouse ear skin and creating a pore within the *stratum corneum* with a diameter of approximately 220 μm. MNs did not penetrate to their full length, with a distinct gap between the skin surface and the MN base plate of approximately 220 μm. The in-skin MN dissolution profile showed biphasic kinetics, with initial rapid reduction in needle height to 25% of the initial depth within the skin for the first 3 min followed by a slower but constant dissolution rate over the next 10 min (Fig. 3). These measurements demonstrate that MN dissolution and delivery of the vaccine formulation occur within a short timeframe following application, and that prolonged maintenance of the applied MN patch is not required.

### 3.2 MNs effectively prime gp140-antigen specific immune responses

**Fig. 4:**
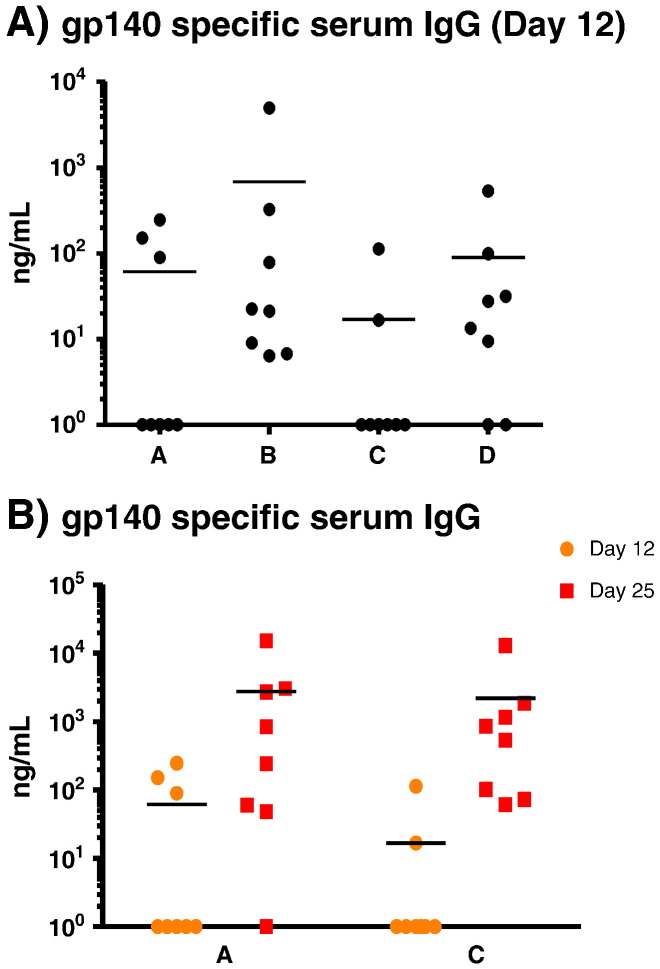
(A) Individual mice gp140 specific serum IgG antibody levels after prime. Non-responders were assigned a value of zero and included in all calculations/statistical analyses; for presentation purposes, these zero values were assigned an arbitrary value of one. The difference between the four groups was not statistically significant and (B) showing that the prime was boosted by vaginal administration (group A) and intranasal administration (group C). Horizontal bars show the mean. Group A: MN prime + Ivag boost; group B: SC prime + SC boost; group C: MN prime + IN boost and group D: MN prime + MN boost.

Mice (groups A, C and D) were primed using MNs containing gp140 and MPLA (Table 1). Priming immunizations were followed by three boost vaccinations at two-week intervals for all study groups. All prime and boost formulations contained 20 μg/dose of MPLA adjuvant. Twelve days after the initial priming application, eleven of 24 animals (46%) elicited gp140-specific serum IgG antibody levels above the level of detection (Fig. 4A). In contrast, 8/8 animals receiving a SC prime (group B) exhibited detectable gp140-specific serum IgG, although, at this early timepoint the serum vaccine antigen-specific IgG levels were not statistically different between the four groups. The first mucosally applied Ivag or IN boost significantly augmented the antigen-specific IgG serum response in MN primed animals, with 15 of the 16 animals (94%) positive for gp140-specific serum IgG; one non-responder remained negative for specific serum IgG throughout the study (Fig. 4B). Mean serum vaccine antigen-specific IgG levels increased ~ 50 folds (vaginal boost) and ~ 130 folds (nasal boost) compared to levels measured post-prime, clearly indicating an anamnestic response and demonstrating that the MN prime had been successful. The mean antigen-specific antibody level in the SC group increased ~ 70 folds after the boost compared to after the prime. However, re-application of the MNs failed to elevate antibody levels after the first boost.

**Fig. 5:**
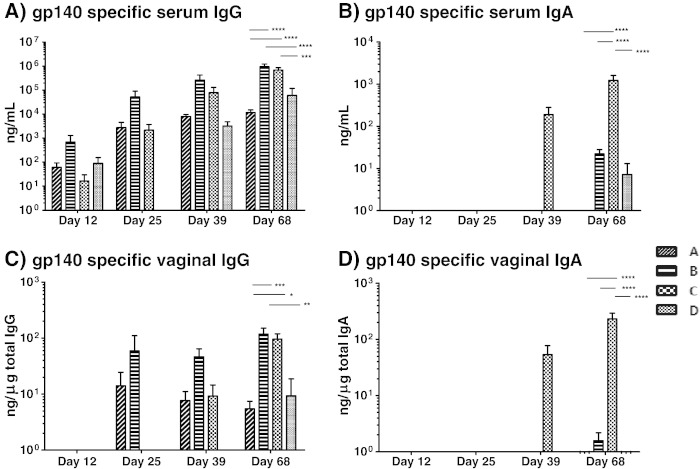
(A) High levels of serum gp140 specific IgG are elicited by all four groups. Non-responders were included in all calculations/statistical analyses and assigned a value of zero. At day 68 groups B and C elicit higher levels of antibody than groups A and D. The difference between groups B and C is not statistically significant. (B) Serum gp140 specific IgA is elicited mainly by group C. The difference between group C and other groups at day 68 is highly significant. (C) Detectable-to-high levels of gp140 specific vaginal IgG are elicited by all groups. The differences at day 68 between groups B and C are not statistically significant. (D) Vaginal gp140 specific IgA is elicited mainly by group C. The difference between group C and other groups at day 68 is highly significant. Note that the scales are logarithmic. Error bars represent SEM. On the graphs * = p < 0.05, ** = p < 0.01, *** = p < 0.001, **** = p < 0.0001 (ANOVA). Differences in antibody levels prior to day 68 are not statistically significant. Group A: MN prime + Ivag boost; group B: SC prime + SC boost; group C: MN prime + IN boost and group D: MN prime + MN boost.

A longitudinal summary of both serum and vaginal mucosal antigen-specific antibody responses showed that all groups responded by producing gp140-specific serum IgG (Fig. 5). Vaginal mucosal antigen-specific IgG was detectable in all groups by the end of the vaccination regime but was already present in groups B (SC) and C (MN + IN) after the respective first boost inoculations. Serum and vaginal mucosal gp140-specific IgA was detected in animals that had received a MN prime followed by an IN boost vaccine (Fig. 5).

### 3.3 MN prime + IN boost regimen elicits similar gp140-specific IgG responses compared to SC alone immunizations

**Fig. 6:**
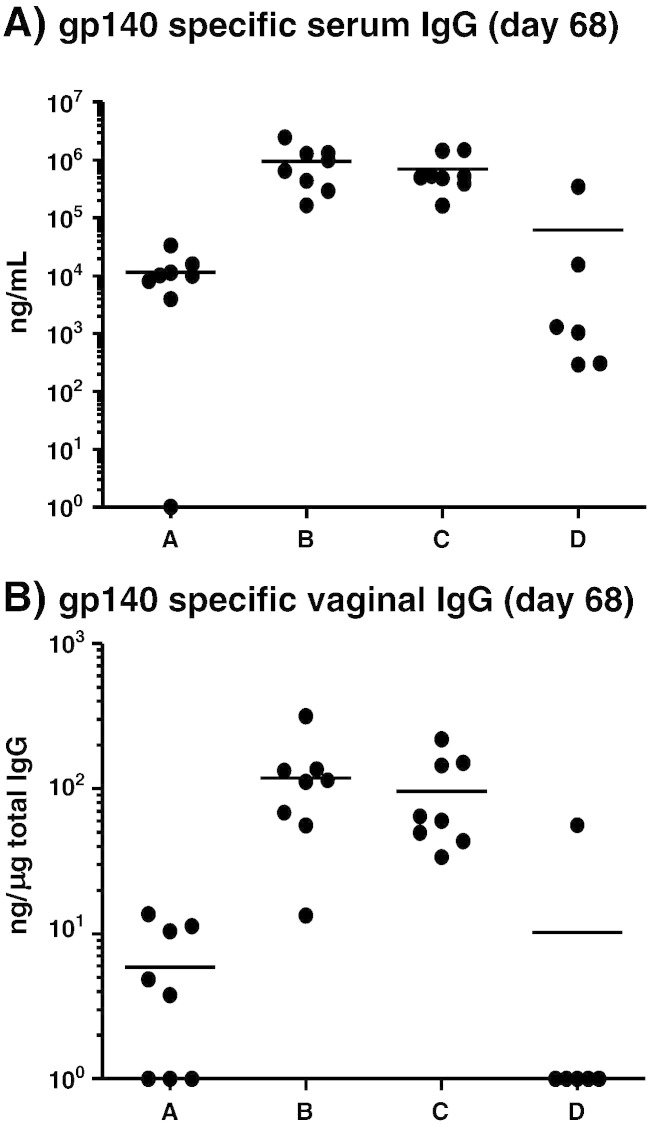
(A) Individual mice gp140 specific serum IgG antibody levels at the end of the study. Non-responders were assigned a value of zero and included in all calculations/statistical analyses; for presentation purposes, these zero values were assigned an arbitrary value of one. Groups B and C elicited the highest antibody levels which were higher than the other two groups (statistically significant; p < 0.0001 for A vs B, A vs C, B vs D and C vs D; A vs D and B vs C are not significant). (B) Individual mice gp140 specific mucosal IgG antibody levels at the end of the study. Groups B and C elicited similar antibody levels. Differences between groups B or C and A or D are statistically significant (p < 0.001 A vs B; p < 0.05 A vs C; p < 0.01 B vs D and A vs D; B vs C and C vs D are not significant). Horizontal bars show the mean. Group A: MN prime + Ivag boost; group B: SC prime + SC boost; group C: MN prime + IN boost and group D: MN prime + MN boost.

At the end of the study, groups B and C (SC and MN + IN, respectively) showed similar levels of antigen-specific IgG in both the serum and mucosal compartments (Fig. 6A, B) with no statistical difference between the groups. However, during the course of the study these groups exhibited different kinetics with respect to the development of a vaginal antigen-specific IgG response. Group A (MN prime + Ivag boost) demonstrated reasonable levels of antigen-specific vaginal IgG after the first boost but this early augmentation of vaginal IgG immune response was not maintained and the level of gp140-specific antibody could not be boosted further via the Ivag route. Conversely, the augmentation of the vaginal responses in animals that received the MN prime followed with IN boosting was significantly delayed and was further boosted by subsequent inoculations (Fig. 5).

### 3.4 MN prime + IN boost regimen uniquely elicits good serum and vaginal mucosal gp140-specific IgA responses

**Fig. 7:**
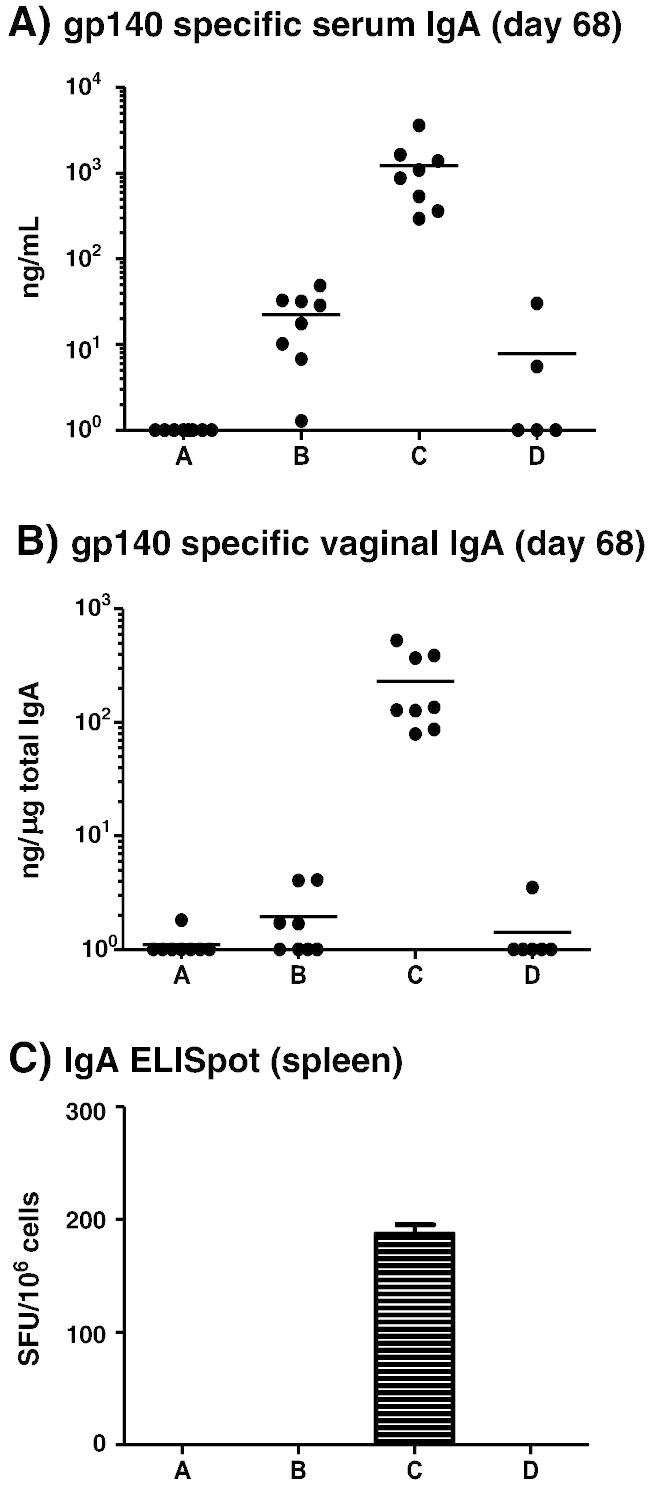
Individual mice (A) gp140 specific serum IgA at the end of the study (p < 0.0001 for A vs C, B vs C and C vs D; A vs B, A vs D and B vs C are not significant) and (B) vaginal IgA antibody levels at the end of the study (p < 0.0001 for A vs C, B vs C and C vs D; A vs B, A vs D and B vs C are not significant). Only mice from group C elicit a robust IgA antibody level both in the serum and vaginal secretions. This difference is highly statistically significant. Horizontal bars show the mean. Group C is the only group showing significant levels of gp140 specific IgA producing cells in the spleen (c, four replicates, error bar shows the SEM). Group A: MN prime + Ivag boost; group B: SC prime + SC boost; group C: MN prime + IN boost and group D: MN prime + MN boost. Non-responders were assigned a value of zero and included in all calculations/statistical analyses; for presentation purposes, these zero values were assigned an arbitrary value of one.

Interestingly, mice receiving the MN prime + IN boost (group C) uniquely developed serum and vaginal vaccine antigen-specific IgA responses after the second boost, with specific antibody levels further augmented after the third boost (Fig. 7A, B). Vaginal boosting of a MN prime failed to elicit any detectable antigen-specific IgA response (group A), while the MN boost group (D) elicited low levels of serum and no detectable mucosal gp140-specific IgA. The SC inoculation only group (B) elicited weak serum and barely detectable mucosal antigen-specific IgA responses after the third boost which were, respectively, ~ 50 folds and ~ 140 folds lower than the MN + IN group (Fig. 7A, B). We next assessed the number of antigen-specific B cells still resident in the spleen at the end of the immunization regime in order to confirm that the antigen-specific serum antibody responses reflected the phenotype of cells elicited by the vaccine modality. An IgA ELISpot confirmed that only the MN + IN group C retained a population of antigen-reactive B lymphocytes in the spleen (Fig. 7C).

### 3.5 MN prime + IN boost vaccine regimen elicited immune responsive cells that were Th1/Th2 balanced but highly primed to respond

**Fig. 8:**
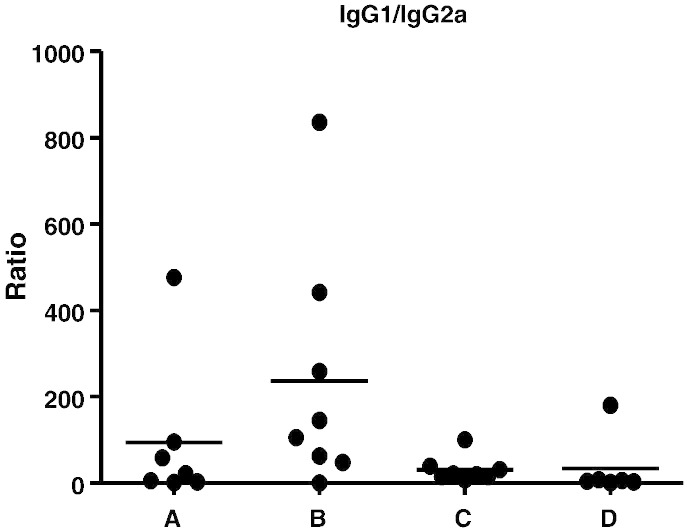
IgG1/IgG2a ratio in serum on day 68. Bars show the mean ratio for each animal. While all groups are Th2 polarized, group B (SC) exhibits a stronger Th2 bias. Group A: MN prime + Ivag boost; group B: SC prime + SC boost; group C: MN prime + IN boost and group D: MN prime + MN boost.

Analysis of the serum antigen-specific IgG1 (Th2) and IgG2a (Th1) antibody isotypes showed that the immune responses elicited in the microneedle-primed groups (A, C and D) were significantly less Th2 polarized than those of the SC control group (Fig. 8). BALB/c mice have a genetic predisposition for a strong Th2 polarization, and the animals from the SC systemic vaccination group (B) clearly demonstrated this bias [18]. The Th2 polarisation bias was less for groups A (MN + Ivag), C (MN + IN) and D (MN only) with the MN + IN group demonstrating a particularly balanced response.

**Fig. 9:**
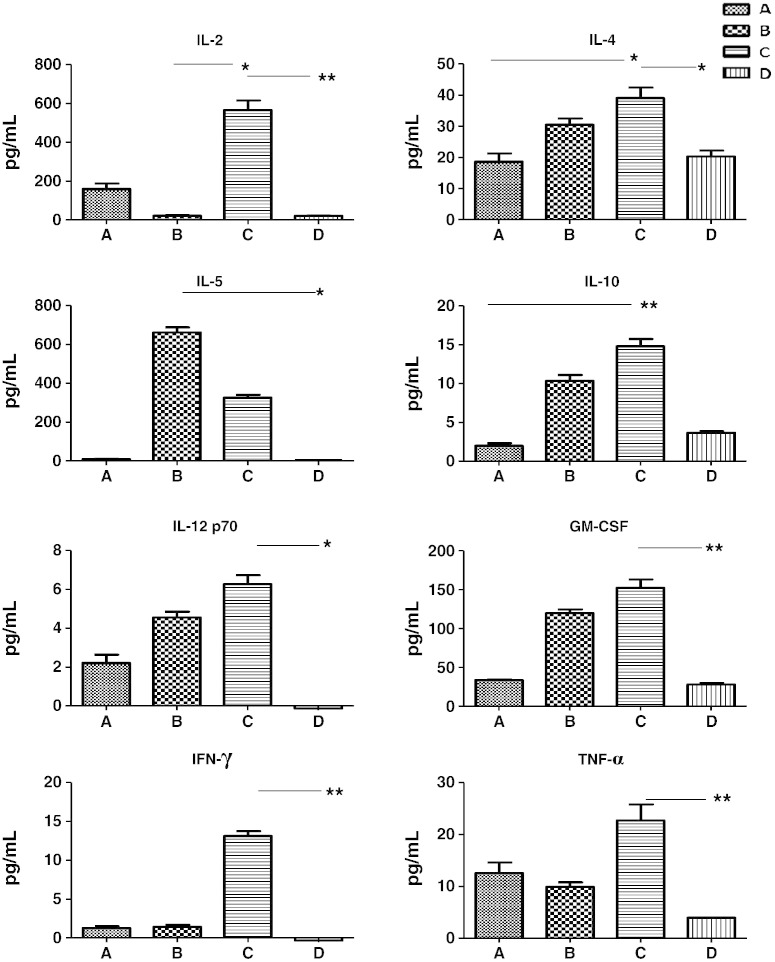
Cytokine profiles produced from the stimulation of spleen cells by gp140. IL-2 and IL-5 were found to be important. IL-5 was dramatically up-regulated by groups B and C while IL-2 was strongly up-regulated by group C. Data represents baseline corrected four replicates from pooled samples on day 68 with error bars representing the SEM. The non-parametric Kruskal–Wallis test was used for statistical analysis. On the graphs * = p < 0.05 and ** = p < 0.01. Concanavalin A was used as a positive control and unstimulated cells were used as a negative control. Unstimulated cells always showed very low levels of cytokines and concanavalin A induced a strong production. The raw data can be seen in Appendix A supporting information (2). Group A: MN prime + Ivag boost; group B: SC prime + SC boost; group C: MN prime + IN boost and group D: MN prime + MN boost.

The cytokine profiles produced upon stimulation of splenocytes harvested from each group at the end of the study were also assessed by Luminex analysis. The SC control (group B) expressed a number of cytokines upon re-stimulation, including high amounts of IL-5 and moderate levels of IL-4, -10 and ‐12 but not IL-2 and IFN-γ (Fig. 9). In contrast, groups A (MN + Ivag) and D (MN alone) did not exhibit any conspicuous cytokine expression profiles. Strikingly, antigen-driven stimulation of group C provided up-regulation of all the cytokines within the Luminex panel indicating that the cells from this vaccination group had a highly inflammatory potential and capacity to respond to the vaccine antigen. It is known that IgA production can be induced and promoted by IL-2, IL-5 and IL-10 augmentation of CD40L stimulated B lymphocytes [19,20]. It is likely significant that very little IgA was produced in group B (that lacked IL-2 but had ↑ IL-5) and that IgA was dramatically increased in group C (↑ IL-5, ↑ IL-2, ↑ IL-10). Since these data correlate well with the serum and mucosal antigen-specific antibody responses, it is our contention that this modality of MN prime and IN boost induced a combination of cytokines that acted either additively or synergistically to result in a striking production of antigen-specific IgA.

## 4 Discussion

We have demonstrated that a novel dissolvable MN array device containing co-formulated vaccine components was able to effectively prime antigen-specific immune responses that were boostable by subsequent topical mucosal vaccination. Measurement of dissolution kinetics showed that the microneedles dissolved rapidly upon application, allowing a rapid release of the vaccine antigen/adjuvant. MN primed animals were boosted via the skin either with MNs or by topical nasal or vaginal application, however the outcomes of these boost inoculations were very different. Intranasal inoculation elicited significant antigen-specific immune responses, with serum and mucosal gp140-specific IgG levels similar to those measured after systemic subcutaneous vaccination. Initial Ivag and MN boosting provided enhancement in antibody levels, but these were not significantly further augmented upon subsequent boosts. These differences are likely due to the immunological inductive potential of the different mucosal tissues. Nasal antigen application provides access to the nasopharynx-associated lymphoid tissue (NALT) with its large population of microfold cells (M cells) and high densities of immunologically responsive cells underneath the follicle-associated epithelium (FAE). Access to both these cell types is able to dramatically enhance the capture, processing and presentation of vaccine antigen [21–23].

Significantly, nasal vaccination also elicited high quantities of vaccine antigen-specific IgA in both the serum and mucosal compartments, which was greatly enhanced over the SC group. This unique augmentation of IgA production potential was also apparent in the numbers of vaccine-antigen specific B lymphocytes resident in the spleen of mice that had received the MN prime + IN boost vaccine. A number of recent publications, particularly the Tudor et al. paper describing the blocking of HIV epithelial transcytosis and neutralization of CD4 T cell infection, have strongly suggested that a good mucosal IgA response is important for prevention of HIV acquisition via mucosal transmission [24,25].

The SC group immune responses showed a high degree of bias toward Th2 polarization, with other groups showing a relatively balanced Th1/Th2 profile. Additional assays to assess whether the cells harvested from the different vaccination groups had skewed polarization profiles were performed to measure the antigen-driven capacity for IL-2, IL-12 p70, IFN-γ and TNF-α (Th1), IL-4, IL-5 and IL-10 (Th2) and GM-CSF expression and secretion. Th1 cytokines induce a cell-mediated immune response with IL-2 playing a role in lymphocyte proliferation, while IFN-γ and TNF-α are pivotal mediators of many anti-viral cellular effector functions. IL-12 plays a key role in the development and maintenance of Th1 skewed immune responses. The Th2 cytokines, IL-4, IL-5 and IL-10, promote the development of antibody producing cells and induction of mucosal antibody-mediated (including IgA) immunity [19,24,26]. In particular, IL-5 is known to be a major promoter of IgA isotype switching and B cell survival, and it is now clear that cytokine combination networks can have potent effects on cellular differentiation. The group receiving the MN prime + IN boost secreted high levels of IL-2, IL-4, IL-5 and IL-10, all of which are known to likely act in synergy to promote the production of IgA [27,28]. It was observed that intranasal boosts with gp140 produced a much stronger immune response than equivalent intravaginal boosts. These results are consistent with a number of previous studies and further confirm the potency of the nasal route of immunisation [10,29]. It seems likely that differences in vaccine-available mucosal surface area, the immunostimulatory potential of the resident APCs, as well as the stability of antigens in the mucosal fluids may have a role to play in explaining this phenomenon.

Additional measures of MN prime + IN boost vaccine efficacy could include neutralisation assays. However, these require larger sample volumes than those obtained from mice in the current investigation; further experiments in another larger species, such as rabbits or macaques, are warranted. The nature of the HIV CN54gp140 vaccine antigen after its formulation within the MNs should also be fully assessed and defined, though our current experiments suggest good stability and conformational integrity. Additional studies are planned to assess the specific roles and contribution of MN and IN boosts to the overall immunological picture. These data are important corroborating elements that would enable a fuller understanding of vaccine device elicited immune responses.

## 5 Conclusion

A novel microneedle prime + IN boost vaccination modality elicited an IgG antibody and lymphocyte proliferation response similar to a systemic subcutaneous regimen. Furthermore, this regimen also elicited high-level antigen-specific vaginal IgA response, an important feature of an effective HIV vaccine, that will also likely require a CD8 + CTL component not addressed within this study. In addition, this novel polymeric microneedle system offers the prospect of a pain-free vaccination method that avoids injection-associated infection and is amenable to mass immunization without the need for trained medical personnel. Since the antigen and adjuvant are formulated in the solid state, stability at elevated temperatures and relative humidities is likely to be greatly enhanced provided that the system is stored in a moisture-impermeable packaging. Although HIV gp140 was used in this study, the microneedle technology platform may be readily applied to any vaccine antigen candidate with the potential to enhance immune responses to any mucosally acquired pathogen. Once fully developed, this delivery system would offer significant advantages for vaccination programmes in a developing world context.

## Figures and Tables

**Table 1:** Administration schedule for the four groups in the study.

Group	Prime day 0	3 × boost (days 14, 28, 42)
A	MN — 10 μg gp140 + 20 μg MPLA^a^	Ivag — 10 μg gp140 + 20 μg MPLA
B	SC — 10 μg gp140 + 20 μg MPLA	SC — 10 μg gp140 and 20 μg MPLA
C	MN — 10 μg gp140 + 20 μg MPLA	IN — 10 μg gp140 + 20 μg MPLA
D	MN — 10 μg gp140 + 20 μg MPLA	MN — 10 μg gp140 + 20 μg MPLA

SC administration volume was 100 μL in PBS, and the IN and Ivag administration volume was 25 μL in PBS.

a MPLA stock solution (10 mg/mL) was prepared in dimethyl sulphoxide (DMSO) and used for all experiments.

**Table 2:** OCT assessment of MN penetration following manual application to the mouse ear in vivo. (Means ± SD, n = 15).

MN penetration depth (μm)	Pore width (μm)	Base plate/SC distance (μm)
393.64 ± 7.45	218.42 ± 4.79	223.30 ± 5.62

## References

[1] Lewis D.J., Fraser C.A., Mahmoud A.N., Wiggins R.C., Woodrow M., Cope A., Cai C., Giemza R., Jeffs S.A., Manoussaka M., Cole T., Cranage M.P., Shattock R.J., Lacey C.J. (2011). Phase I randomised clinical trial of an HIV-1_CN54_, clade C, trimeric envelope vaccine candidate delivered vaginally. PLoS One.

[2] Donnelly L., Curran R.M., Tregoning J.S., McKay P.F., Cole T., Morrow R.J., Kett V.L., Andrews G.P., Woolfson A.D., Malcolm R.K., Shattock R.J. (2011). Intravaginal immunization using the recombinant HIV-1 clade-C trimeric envelope glycoprotein CN54gp140 formulated within lyophilized solid dosage forms. Vaccine.

[3] Prausnitz M.R., Mikszta J.A., Cormier M., Andrianov A.K. (2009). Microneedle-based vaccines. Curr. Top. Microbiol. Immunol..

[4] Kendall M. (2006). Engineering of needle-free physical methods to target epidermal cells for DNA vaccination. Vaccine.

[5] Huang C.M. (2007). Topical vaccination: the skin as a unique portal to adaptive immune responses. Semin. Immunopathol..

[6] Lambert P.H., Laurent P.E. (2008). Intradermal vaccine delivery: will new delivery systems transform vaccine administration?. Vaccine.

[7] Alarcon J.B., Hartley A.W., Harvey N.G., Mikszta J.A. (2007). Preclinical evaluation of microneedle technology for intradermal delivery of influenza vaccines. Clin. Vaccine Immunol..

[8] Matriano J.A., Cormier M., Johnson J., Young W.A., Buttery M., Nyam K., Daddona P.E. (2002). Macroflux (R) microprojection array patch technology: a new and efficient approach for intracutaneous immunization. Pharm. Res..

[9] Sullivan S.P., Koutsonanos D.G., Del Pilar Martin M., Lee J.W., Zarnitsyn V., Choi S.O., Murthy N., Compans R.W., Skountzou I., Prausnitz M.R. (2010). Dissolving polymer microneedle patches for influenza vaccination. Nat. Med..

[10] Arias M.A., Loxley A., Eatmon C., Van Roey G., Fairhurst D., Mitchnick M., Dash P., Cole T., Wegmann F., Sattentau Q., Shattock R. (2011). Carnauba wax nanoparticles enhance strong systemic and mucosal cellular and humoral immune responses to HIV-gp140 antigen. Vaccine.

[11] Curran R.M., Donnelly L., Morrow R.J., Fraser C., Andrews G., Cranage M., Malcolm R.K., Shattock R.J., Woolfson A.D. (2009). Vaginal delivery of the recombinant HIV-1 clade-C trimeric gp140 envelope protein CN54gp140 within novel rheologically structured vehicles elicits specific immune responses. Vaccine.

[12] Cranage M.P., Fraser C.A., Cope A., Mckay P.F., Seaman M.S., Cole T., Mahmoud A.N., Hall J., Giles E., Voss G., Page M., Almond N., Shattock R.J. (2011). Antibody responses after intravaginal immunisation with trimeric HIV-1(CN54) clade C gp140 in Carbopol gel are augmented by systemic priming or boosting with an adjuvanted formulation. Vaccine.

[13] Cranage M.P., Fraser C.A., Stevens Z., Huting J., Chang M., Jeffs S.A., Seaman M.S., Cope A., Cole T., Shattock R.J. (2010). Repeated vaginal administration of trimeric HIV-1 clade C gp140 induces serum and mucosal antibody responses. Mucosal Immunol..

[14] Su L., Graf M., Zhang Y., von Briesen H., Xing H., Kostler J., Melzl H., Wolf H., Shao Y., Wagner R. (2000). Characterization of a virtually full-length human immunodeficiency virus type 1 genome of a prevalent intersubtype (C/B′) recombinant strain in China. J. Virol..

[15] Rodenburg C.M., Li Y.Y., Trask S.A., Chen Y., Decker J., Robertson D.L., Kalish M.L., Shaw G.M., Allen S., Hahn B.H., Gao F. (2001). Near full-length clones and reference sequences for subtype C isolates of HIV type 1 from three different continents. AIDS Res. Hum. Retroviruses.

[16] Donnelly R.F., Majithiya R., Thakur R.R.S., Morrow D.I.J., Garland M.J., Demir Y.K., Migalska K., Ryan E., Gillen D., Scott C.J., Woolfson A.D. (2011). Design, optimization and characterisation of polymeric microneedle arrays prepared by a novel laser-based micromoulding technique. Pharm. Res..

[17] Donnelly R.F., Garland M.J., Morrow D.I.J., Migalska K., Thakur R.R.S., Majithiya R., Woolfson A.D. (2010). Optical coherence tomography is a valuable tool in the study of the effects of microneedle geometry on skin penetration characteristics and in-skin dissolution. J. Control. Release.

[18] Finnegan A., Mikecz K., Tao P., Glant T.T. (1999). Proteoglycan (aggrecan)-induced arthritis in BALB/c mice is a Th1-type disease regulated by Th2 cytokines. J. Immunol..

[19] Kohtaro F., McGhee J.R., Mesteky J., Lamm M.E., McGhee J.R., Bienenstock J., Mayer L., Strober W. (2005). TH1/TH2/TH3 cells for regulation of mucosal immunity, tolerance and inflammation. Mucosal Immunology.

[20] Cerutti A. (2008). The regulation of IgA class switching. Nat. Rev. Immunol..

[21] Johansson E., Rask C., Fredriksson M., Eriksson K., Czerkinsky C., Holmgren J. (1998). Antibodies and antibody-secreting cells in the female genital tract after vaginal or intranasal immunization with cholera toxin B subunit or conjugates. Infect. Immun..

[22] Mestecky J., Russell M.W. (2000). Induction of mucosal immune responses in the human genital tract. FEMS Immunol. Med. Microbiol..

[23] Vajdy M., Singh M., Kazzaz J., Soenawan E., Ugozzoli M., Zhou F., Srivastava I., Bin Q., Barnett S., Donnelly J., Luciw P., Adamson L., Montefiori D., O'Hagan D.T. (2004). Mucosal and systemic anti-HIV responses in rhesus macaques following combinations of intranasal and parenteral immunizations. AIDS Res. Hum. Retroviruses.

[24] Kiyono H., Miller C.J., Yichen L., Lehner T., Cranage M., Yung T.H., Kawabata S., Marthas M., Roberts B., Nedrud J.G., Lamm M.E., Bergmeier L., Brookes R., Tao L., McGhee J.R. (1995). The common mucosal immune system for the reproductive tract: basic principles applied toward an AIDS vaccine. Adv. Drug Deliv. Rev..

[25] Tudor D., Derrien M., Diomede L., Drillet A.S., Houimel M., Moog C., Reynes J.M., Lopalco L., Bomsel M. (2009). HIV-1 gp41-specific monoclonal mucosal IgAs derived from highly exposed but IgG-seronegative individuals block HIV-1 epithelial transcytosis and neutralize CD4(+) cell infection: an IgA gene and functional analysis. Mucosal Immunol..

[26] Kidd P. (2003). Th1/Th2 balance: the hypothesis, its limitations, and implications for health and disease. Altern. Med. Rev..

[27] Eckmann L., Morzycka-Wroblewska E., Smith J.R., Kagnoff M.F. (1992). Cytokine-induced differentiation of IgA B cells: studies using an IgA expressing B-cell lymphoma. Immunology.

[28] Brière F., Bridon J.M., Chevet D., Souillet G., Bienvenu F., Guret C., Martinez-Valdez H., Banchereau J. (1994). Interleukin 10 induces B lymphocytes from IgA-deficient patients to secrete IgA. J. Clin. Invest..

[29] Sakaue G., Hiroi T., Nakagawa Y., Someya K., Iwatani K., Sawa Y., Takahashi H., Honda M., Kunisawa J., Kiyono H. (2003). HIV mucosal vaccine: nasal immunization with gp160-encapsulated hemagglutinating virus of Japan-liposome induces antigen-specific CTLs and neutralizing antibody responses. J.Immunol..

