# Supplementary material for: Microneedle mediated intradermal delivery of adjuvanted recombinant HIV-1 CN54gp140 effectively primes mucosal boost inoculations
Source: J Control Release. 2012 Sep 28;162(3):529–37. doi: 10.1016/j.jconrel.2012.07.039 (PMC3778941; doi:10.1016/j.jconrel.2012.07.039)
Supplement: Supplementary file 1 — Supplementary materials. [file mmc1.pdf]

Supporting Information

1. Two mice from group D died during the administration of the first boost. These mice were autopsied. The deaths were found to be procedural and not attributed to the formulation.
2. The complete data showing cytokine production after stimulation of spleen cells with either gp140 or concanavalin A or with no stimulation (figure 10).

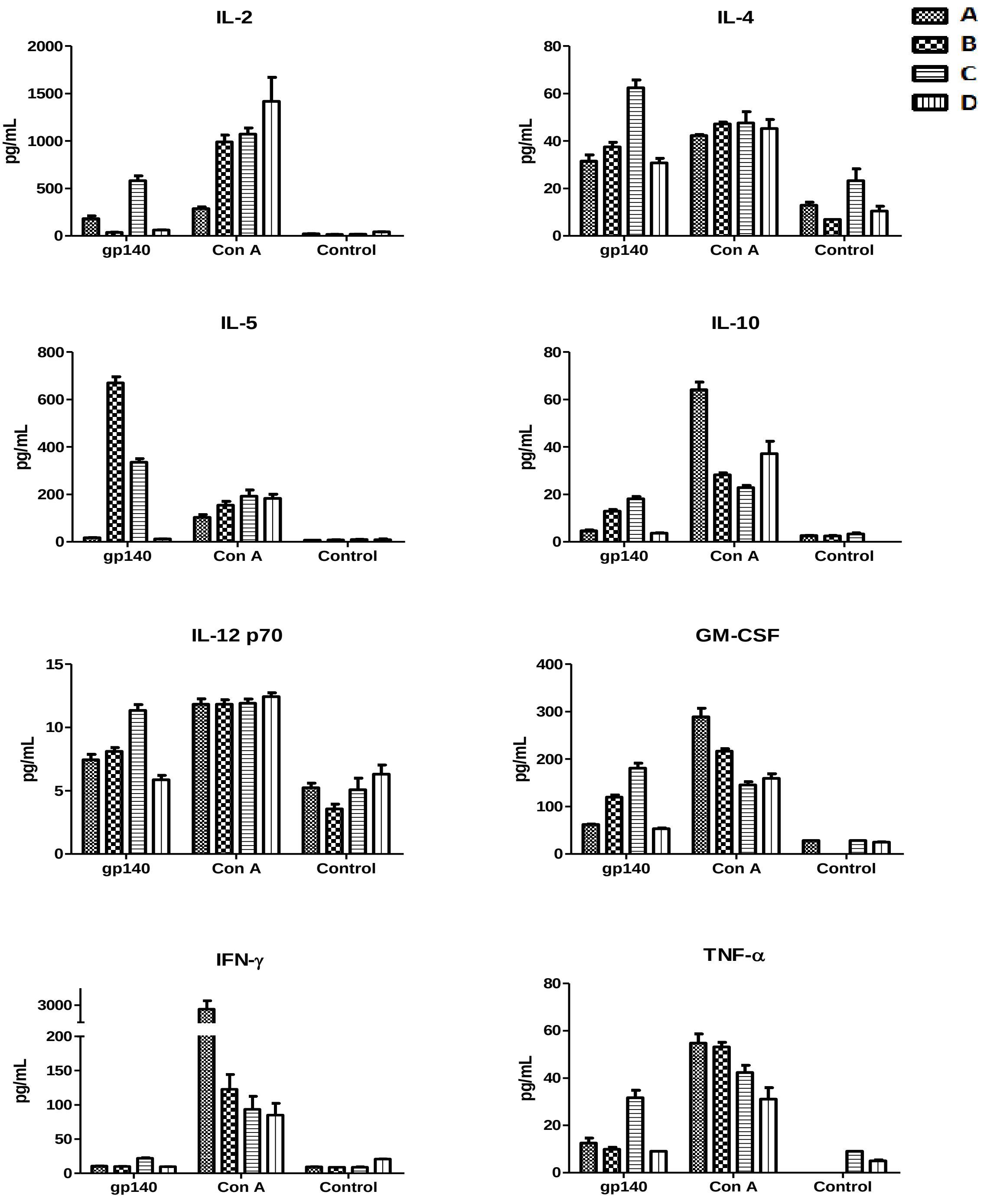

Figure 10: Cytokine profiles produced from the stimulation of spleen cells by gp140. IL-5 was dramatically upregulated by group B. Con A was used as a positive control and unstimulated cells as a negative (control). Group A: MN Prime + Ivag boost; Group B: SC prime + SC boost; Group C: MN prime + IN boost and Group D: MN prime + MN boost.
